# Resource Availability Drives Responses of Soil Microbial Communities to Short-term Precipitation and Nitrogen Addition in a Desert Shrubland

**DOI:** 10.3389/fmicb.2018.00186

**Published:** 2018-02-09

**Authors:** Weiwei She, Yuxuan Bai, Yuqing Zhang, Shugao Qin, Wei Feng, Yanfei Sun, Jing Zheng, Bin Wu

**Affiliations:** ^1^Yanchi Research Station, School of Soil and Water Conservation, Beijing Forestry University, Beijing, China; ^2^Key Laboratory of State Forestry Administration on Soil and Water Conservation, Beijing Forestry University, Beijing, China; ^3^Engineering Research Center of Forestry Ecological Engineering, Ministry of Education, Beijing Forestry University, Beijing, China

**Keywords:** copiotrophic/oligotrophic, global environmental changes, microbial diversity and community composition, nitrogen deposition, precipitation changes, soil bacteria and fungi, stressful environment, stress-tolerant

## Abstract

Desert microbes are expected to be substantially sensitive to global environmental changes, such as precipitation changes and elevated nitrogen deposition. However, the effects of precipitation changes and nitrogen enrichment on their diversity and community composition remain poorly understood. We conducted a field experiment over 2 years with multi-level precipitation and nitrogen addition in a desert shrubland of northern China, to examine the responses of soil bacteria and fungi in terms of diversity and community composition and to explore the roles of plant and soil factors in structuring microbial communities. Water addition significantly increased soil bacterial diversity and altered the community composition by increasing the relative abundances of stress-tolerant (dormant) taxa (e.g., *Acidobacteria* and *Planctomycetes*); however, nitrogen addition had no substantial effects. Increased precipitation and nitrogen did not impact soil fungal diversity, but significantly shifted the fungal community composition. Specifically, water addition reduced the relative abundances of drought-tolerant taxa (e.g., the orders *Pezizales, Verrucariales*, and *Agaricales*), whereas nitrogen enrichment decreased those of oligotrophic taxa (e.g., the orders *Agaricales* and *Sordariales*). Shifts in microbial community composition under water and nitrogen addition occurred primarily through changing resource availability rather than plant community. Our results suggest that water and nitrogen addition affected desert microbes in different ways, with watering shifting stress-tolerant traits and fertilization altering copiotrophic/oligotrophic traits of the microbial communities. These findings highlight the importance of resource availability in driving the desert microbial responses to short-term environmental changes.

## Introduction

Deserts occupy approximately one-third of the Earth’s land surface (Laity, 2009) and are currently experiencing widespread global environmental changes, including precipitation changes and elevated nitrogen deposition (Galloway et al., 2008; Maestre et al., 2012). As desert ecosystems are usually characterized by stressful conditions of low water and nutrient availability (Noy-Meir, 1973; Makhalanyane et al., 2015), they are expected to be substantially more sensitive to global environmental changes than other ecosystems (Bobbink et al., 2010; Maestre et al., 2016). Soil microbial diversity and community composition in desert biomes have been shown to differ remarkably from those in non-desert biomes (Fierer et al., 2012), suggesting that their responses to environmental changes might also be distinct. However, the impacts of environmental changes on desert microbial communities are not well-understood.

Soil microbes play a crucial role in biogeochemical cycles, and thus their responses to environmental changes can provide feedback to influence plant communities and climate systems (Nie et al., 2013; Wei et al., 2013). Recent high-throughput sequencing data have shown that both precipitation changes and nitrogen deposition can alter the microbial community composition and diversity (Evans and Wallenstein, 2014; Leff et al., 2015; Li et al., 2016; Zeng et al., 2016). In general, nitrogen addition tends to decrease microbial diversity, increase the relative abundance of copiotrophic (i.e., fast-growing, low carbon-use efficiency) taxa (e.g., *Proteobacteria* and *Bacteroidetes*), and reduce that of oligotrophic (i.e., slow-growing, high carbon-use efficiency) taxa (e.g., *Acidobacteria* and *Basidiomycota*) (Fierer et al., 2007; Ramirez et al., 2012; Leff et al., 2015; Ho et al., 2017). It is generally hypothesized that precipitation enrichment, similarly to nitrogen addition, could favor copiotrophic taxa over oligotrophic taxa due to water-induced increases in nitrogen availability (Li et al., 2017b); however, results of the impacts of precipitation changes on microbial communities are inconsistent. Data from many field experiments have suggested that background precipitation variability (e.g., seasonal or interannual variability) more strongly shapes the microbial community composition than does the direct effect of experimental precipitation treatments (Cregger et al., 2012; Gutknecht et al., 2012; Curiel Yuste et al., 2014). Data from other studies have indicated that indirect environmental factors (e.g., the plant community) that shift under precipitation changes might play a larger role in determining microbial community composition than direct changes to soil moisture (Evans et al., 2014; Li et al., 2016). Water-induced indirect effects on microbial communities might be ecosystem-specific, which may contribute to previous inconsistent results among different precipitation-manipulation experiments. Collectively, these previous findings suggest that nitrogen addition consistently impacts microbial communities, whereas the inconsistent responses of microbes to precipitation changes may result from ecosystem-specific background/history precipitation regimes and/or water-induced indirect environmental factors.

Precipitation changes and nitrogen addition not only directly affect microbial physiology but also indirectly influence microbial abundances by changing plant and soil properties (Chen et al., 2015; Li et al., 2017b). Water and nitrogen availability can change the quantity and quality of plant residua (e.g., root exudates and litter), which represent major resource inputs to soil (Ren et al., 2015; Liu et al., 2016). These changes can impact microbial structure and function (Wardle et al., 2006; Bardgett and van der Putten, 2014). Several lines of empirical evidence have shown that nitrogen-induced soil acidification exerts native effects on microbes (Chen et al., 2015; Zeng et al., 2016), while precipitation increment can dampen these effects by increasing the soil pH (Zhang et al., 2014; Li et al., 2016). Similarly, precipitation and nitrogen enrichment have counteractive effects on plant species richness, with water-induced increases and nitrogen-induced declines in plant diversity (Xu Z. et al., 2015). Plant diversity has been documented as one of the major drivers in structuring microbial communities (Prober et al., 2015; Chen et al., 2017; Li et al., 2017a), suggesting that increased precipitation might alleviate the effects of nitrogen-induced loss of plant richness on microbial communities through increasing plant diversity. Collectively, previous findings have shown the very complex nature of the interactive influences of precipitation changes and nitrogen addition on microbial communities, which occur via the alteration of plant communities and soil properties.

To examine the effects of precipitation and nitrogen addition on soil microbial communities in desert ecosystems, we conducted a field experiment by varying precipitation and nitrogen addition to a desert shrubland in the Mu Us Desert of northern China. Our main goals were (i) to test how soil bacterial and fungal communities respond to increased precipitation and nitrogen and (ii) to identify the relative roles of the direct and indirect effects of water and nitrogen addition on microbial communities, thereby advancing a potential mechanistic understanding of the responses of desert microbial communities to global environmental changes.

## Materials and Methods

### Study Site and Experimental Design

This study was conducted at the Yanchi Research Station (37°04′–38°10′ N, 106°30′–107°41′ E, elevation 1,530 m above sea level), which is located on the southwestern edge of the Mu Us Desert in Ningxia, China. This region is characterized by a semiarid continental monsoon climate with an average annual temperature of 8.1°C and a mean annual precipitation of 284.8 mm (1955–2013). Approximately 80% of the precipitation occurs from May to September. The mean annual potential evapotranspiration is 2,024 mm (Jia et al., 2016). The soil type is quartisamment based on the US Soil Taxonomy (Gao et al., 2014). The dominant shrub species in this region is *Artemisia ordosica*, although sparse shrubs (*Hedysarum mongolicum, Salix psammophila*, and *Caragana korshinskii*) and the grass *Agropyron cristatum* are also indigenous to the region (She et al., 2015). The study was conducted in a fenced area, in which grazing has been prohibited since the late 1990s and vegetation was allowed to recover for over a decade (Sun et al., 2016).

A two-factor field experiment performed with constant precipitation and nitrogen addition was established in October 2014. There were three precipitation levels (W0: ambient; W20: ambient + 20%; and W40: ambient + 40%) and two nitrogen levels (N0: ambient, 0 kg N ha^-1^ yr^-1^ and N60: fertilization, 60 kg N ha^-1^ yr^-1^), resulting in a total of six treatments (including all permutations of both factors), each with four replications. Twenty-four 5 m × 5 m plots were laid out in a randomized block design (four blocks with six treatment plots within each block). Plots were separated by a 1 m wide buffer zone.

The amount of added precipitation was determined by referring to the long-term mean annual precipitation in this area (1955–2013: 284.8 mm). Specifically, the W20 and W40 treatment plots received 20% (∼56 mm) and 40% (∼112 mm) of supplementary precipitation, respectively. According to the magnitude and distribution of long-term mean monthly precipitation in this area (see details in She et al., 2016), water was applied with a sprinkler irrigation system as nine equal applications during the growing season (May–September), three times in July and August and once in May, June, and September. Water was added following natural rainfall events to avoid altering the precipitation regime. In this study, we defined annual precipitation as the water-year precipitation received between October 1 and September 30 in the following year. Ambient water-year precipitation was 288 and 369 mm in 2015 and 2016, respectively.

Fertilization treatments involved five equal applications of NH_4_NO_3_ solution at the beginning of each month during the growing season, corresponding to a total fertilization rate of 60 kg N ha^-1^ yr^-1^ for the N60 treatment. For each fertilization event, NH_4_NO_3_ (analytical grade) was weighed and dissolved in 10 L tap water. The same amount of water, equivalent to a 2 mm precipitation, was applied to the nitrogen control plots. The rate of nitrogen addition in the N60 treatment was nearly fivefold greater than the background deposition rate in the study site (12 kg N ha^-1^ yr^-1^; She, unpublished data), but was well within the range of current deposition rates in northern China (an average value of 56.2 kg N ha^-1^ yr^-1^; Xu W. et al., 2015).

### Plant and Soil Measurements

In mid-September 2016, herbaceous vegetation structure (i.e., plant density, height, and coverage) was investigated using two permanent 1 m × 1 m quadrats in each plot. One of the quadrats was placed under a shrub canopy, and another was placed in the surrounding interspace between shrubs. The number, height, and coverage of shrub species were estimated in each 5 m × 5 m plot. Using these measurements, the plant Shannon–Wiener (SW) index was calculated as the relative abundance of each plant species. Herbaceous aboveground biomass was measured by randomly clipping a 1 m × 1 m quadrat within each plot. The harvested plants were sorted by species, oven-dried at 75°C for 48 h, and weighed. We used herbaceous aboveground biomass measurements to estimate the herbaceous aboveground net primary productivity (ANPP) and classified herbs into two functional groups according to their life forms, including perennial herbs (PH) and annuals (AS). The shrub ANPP was estimated by using an improved method (see details in She et al., 2016) based on the length and number of current-year plant twigs.

After harvesting plant biomass, soil samples were collected to depths of 0-20 cm from the interspaces between shrubs. For each plot, three sample cores were randomly collected using a 3.8 cm diameter soil auger and then composited into a single sample. All collected samples were transported immediately to our laboratory. Fresh samples were sieved through 2 mm screens and stored at 4°C before chemical analysis and at -80°C before soil DNA extraction. All soil samples were separated into two portions. One portion was maintained fresh for measurements of soil moisture and dissolvable inorganic nitrogen (nitrate and ammonium). The other portion was air-dried for determinations of soil pH, soil organic carbon (SOC), soil total nitrogen (STN), and soil total phosphorous (STP). Soil moisture was determined after oven-drying at 105°C for 24 h. Soil nitrate and ammonium were extracted with 2 M KCl and analyzed by dual-wavelength ultraviolet spectrophotometry and indophenol blue colorimetry, respectively. Soil pH was measured in a soil/water (1:2.5) suspension. SOC was measured using the potassium dichromate oxidation method. STN was analyzed by the micro-Kjeldahl method. STP was determined by the Mo–Sb anti-spectrophotometric method.

### Soil DNA Extraction, Amplification, and Sequencing

Genomic DNA was isolated from 0.25 g of each soil sample using the MoBio PowerSoil DNA Isolation Kit (MoBio Laboratories, Inc., United States) following the manufacturer’s instructions. DNA concentrations and purities were assessed by 1% agarose gel electrophoresis. DNA samples were diluted to 1 ng μL^-1^ in sterile water.

The V4 hypervariable region of bacterial 16S rRNA was amplified from bacterial DNA samples with a barcoded primer set, including primers 515F (5′-GTG CCA GCM GCC GCG GTA A-3′) and 806R (5′-GGA CTA CHV GGG TWT CTA AT-3′). The V4 hypervariable region of fungal 18S rRNA was amplified using a barcoded primer set, including primers 528F (5′-GCG GTA ATT CCA GCT CCA A-3′) and 706R (5′-AAT CCR AGA ATT TCA CCT CT-3′). All PCR procedures were performed in 30 μL reaction mixtures containing 15 μL of Phusion^®^ High-Fidelity PCR Master Mix with GC Buffer (New England BioLabs, Inc., United States), 0.2 μM of each primer, and approximately 10 ng template DNA. Thermal cycling involved an initial denaturation at 98°C for 1 min, followed by 30 cycles of denaturation at 98°C for 10 s, annealing at 50°C for 30 s, and elongation at 72°C for 60 s, with a final extension at 72°C for 5 min.

Polymerase chain reaction products were assessed by 2% agarose gel electrophoresis, and those with a bright main band between 400 and 450 bp were chosen for further experiments. Equal amounts of the PCR product from each sample were pooled and then purified using the GeneJET Gel Extraction Kit (Thermo Scientific, Inc., United States). A sequencing library was generated using the NEB Next^®^ Ultra^TM^ DNA Library Prep Kit for Illumina (New England BioLabs, Inc., United States) following the manufacturer’s instructions, and then sequencing adapters were added to the 5′ ends of the amplicons. The library quality was assessed on the Qubit^®^ 2.0 Fluorometer (Thermo Scientific, Inc., United States) and Agilent Bioanalyzer 2100 system (Agilent Technologies, Inc., United States). Finally, the qualified library was sequenced using an Illumina HiSeq 2500 platform (Illumina, Inc., United States) at Novogene (Beijing, China), producing 250-bp paired-end reads.

### Bioinformatics Processing

Paired-end reads were assigned to samples based on their unique barcodes and were truncated by filtering out the barcode and primer sequences. Paired-end reads from the original amplicon were merged using FLASH software (Magoc and Salzberg, 2011) which is a very fast and accurate analysis tool used to merge paired-end reads when the original DNA fragments are shorter than twice the length of reads. The obtained splicing sequences were referred to raw tags. Quality filtering of the raw tags was performed under specific filtering conditions to obtain high-quality clean tags (Bokulich et al., 2013) using QIIME software (Caporaso et al., 2010) as a quality-control process. The clean tags were compared with the reference database using the UCHIME algorithm (Edgar et al., 2011) to detect chimeric sequences (Haas et al., 2011). After removing the chimeric sequences, we obtained effective tags.

Effective tags were clustered into operational taxonomic units (OTUs) at ≥97% sequence similarity with the UPARSE program (Edgar, 2013), and singleton OTUs (with only one read) were removed. A representative sequence from each OTU was selected and annotated for taxonomic information using the Ribosomal Database Project classifier (Wang et al., 2007) against the SILVA Database (Quast et al., 2013). Phylogenetic relationships of different OTUs were conducted using MUSCLE software (Edgar, 2004). OTU abundance tables were constructed using USEARCH software. The relative abundances of species at different taxonomic levels were calculated using the OTU abundance tables. To rarify all data sets to the same level of sampling effort, OTU abundance tables were rarefied to 45,469 bacterial 16S rRNA sequences and 3,138 fungal 18S rRNA sequences (the minimum number of sequences for a sample) for each soil sample. Further analysis of alpha diversity and beta diversity were performed based on these rarefied OTU tables. Finally, microbial diversity was assessed based on the observed species (species richness) and SW index, and Bray–Curtis dissimilarities were used to assess differences in microbial community composition among treatment groups.

All raw sequence reads generated in this study were archived in the Sequence Read Archive database of the National Center for Biotechnology Information under accession number SRP126812.

### Statistical Analyses

The Shapiro–Wilk test was conducted to examine the normality of data that were used for analysis of variance (ANOVA). Data that did not meet the assumption of normality were log/sqrt-transformed prior to analyses to normalize their distributions. Two-way ANOVA was performed to test the impacts of water, nitrogen addition, and their interactions on plant ANPP and diversity, soil chemical properties, microbial diversity, and the relative abundances of dominant microbial taxa. One-way ANOVA with Duncan’s multiple-range tests was performed to compare the effects of watering on each response variable at each nitrogen-addition rate and to compare the effects of nitrogen at each water-treatment level. To estimate the effect sizes of water or nitrogen treatment on the relative abundances of the dominant microbial taxa, the response ratio was calculated as ln(*X_ij_* / *X_ic_*), where *X_ij_* is the observed value for variable *i* in each experimental plot *j* and *X_ic_* is the mean value of the variable *i* in the control treatment (Byrne et al., 2017). One-sample *t*-test was applied to determine whether each response ratio was significantly different from zero. Differences in microbial community composition (Bray–Curtis dissimilarities) among the water- and nitrogen-treatment groups were assessed by permutational multi-variate ANOVA (PERMANOVA) and visualized using principal coordinate analysis (PCoA). Spearman’s rank correlation analysis (Mantel test) was used to test the relationships between plant/soil variables and the microbial community composition. Stepwise regression analysis was conducted to explore the multi-variate effects of plant and soil factors on the microbial diversity and relative abundances of dominant microbial phyla. Prior to performing stepwise regression, environmental factors that showed a high collinearity with other factors (*r* > 0.6) were removed, and other factors (plant SW, shrub ANPP, PH ANPP, AS ANPP, soil moisture, dissolvable inorganic nitrogen (DIN), and STN) were retained.

We conducted structural equation modeling (SEM) to specifically test the direct and indirect effects of water and nitrogen addition on the composition of soil microbial communities (as assessed by PCo1 of the Bray–Curtis dissimilarity matrix). Prior to performing SEM analysis, we hypothesized that increased precipitation and nitrogen would impact the bacterial/fungal communities, directly by increasing water and nitrogen availability, or indirectly through changing plant ANPP, based on our ANOVA, Mantel test, and stepwise regression results. Subsequently, *a priori* model of the above hypothetical relationships was constructed (Supplementary Figure S1). Thus, the pairwise correlations among these environmental factors, including the water- and nitrogen-addition rates, soil moisture, DIN, shrub ANPP, PH ANPP, AS ANPP, and bacterial/fungal PCo1 values, were examined by Spearman’s rank correlation analysis. The matrix of *R* values derived from the Spearman’s correlation analysis was retained to conduct SEM. The data matrix was fitted to the model using the maximum likelihood estimation method (Grace, 2006). The overall goodness-of-fit of our model was characterized by a non-significant chi-square test (*P* > 0.05), low Akaike information criteria (AIC), low root-mean-square error of approximation (RMSEA < 0.05 and *P* > 0.1), and a comparative fit index (CFI) > 0.95. The final model was improved by removing relationships between observed variables from *a priori* models, according to these indices.

All analyses and figures were performed with R software, version 3.3.1 (R Core Team, 2016). Statistical significance was determined at a level of *P* ≤ 0.05. We used the “vegdist,” “adonis,” and “capscale” functions in the vegan package (Oksanen et al., 2013) for computing the Bray–Curtis dissimilarity, conducting PERMANOVA and Mantel test, and implementing PCoA, respectively. We employed the “stepAIC” function in the MASS package (Venables and Ripley, 2002) to conduct stepwise regression analysis, the psych package (Revelle, 2016) to construct a Spearman’s rank correlation matrix, the lavaan package (Yves, 2012) for SEM, and the ggplot2 package (Wickham, 2009) for generating graphs.

## Results

### Plant and Soil Properties

Water addition significantly increased plant SW, but did not affect plant ANPP (Supplementary Table S1). Plant SW increased from 1.14 ± 0.05 (W0 treatment) to 1.23 ± 0.04 (W20) and 1.40 ± 0.05 (W40), amounting to average increment rates of 7.89 and 22.81%, respectively. Nitrogen addition significantly enhanced PH ANPP, but did not alter plant SW, shrub ANPP, and AS ANPP (Supplementary Table S1). Nitrogen-induced increase in PH ANPP under N60 treatment (58.13 ± 14.66 g m^-2^) was 178.13% higher than that under ambient treatment (N0, 20.90 ± 6.15 g m^-2^). No significant interactive effects of water and nitrogen addition were found among any plant properties.

Water and nitrogen enrichment significantly influenced soil moisture and nitrogen availability, but did not affect soil pH, SOC, STN, or STP (Supplementary Table S1). Soil moisture significantly increased only in plots with added water, from 4.18 ± 0.27 (W0) to 6.04 ± 0.38 (W20) and 7.53 ± 0.36% (W40), an average increase of 44.50 and 80.14%, respectively. Nitrogen-induced increase in soil nitrogen availability in the plots with fertilization (N60, 2.40 ± 0.17 mg N kg^-1^) was 144.90% higher than that in unfertilized plots (N0, 0.98 ± 0.08 mg N kg^-1^). No significant interactive effects were detected among any soil properties.

### Diversity and Composition of Microbial Communities

**FIGURE 1:**
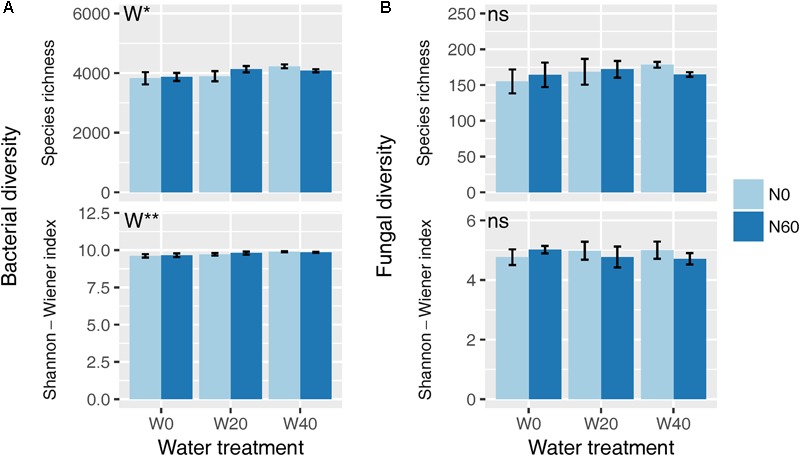
Effects of water and nitrogen addition on soil bacterial **(A)** and fungal **(B)** diversity. ^∗^*P* < 0.05, ^∗∗^*P* < 0.01; ns, not significant.

**FIGURE 2:**
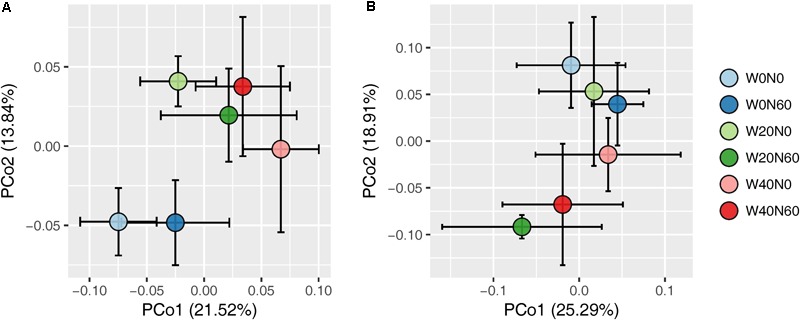
Principal coordinate analysis of soil bacterial **(A)** and fungal **(B)** community differences (Bray–Curtis dissimilarities) following different water and nitrogen treatments.

Soil bacterial species richness and SW index showed positive responses to water addition, but were unresponsive to nitrogen addition and water–nitrogen interactions (**Figure 1A** and Supplementary Table S1). Relative to ambient treatment, water addition increased bacterial species richness and SW index by 4.28-7.91% and 1.35-2.50%, respectively. Shifts in soil bacterial community composition were detected under different water treatments (PERMANOVA, *F* = 1.800, *P* = 0.005), but not under nitrogen treatments (PERMANOVA, *F* = 0.826, *P* = 0.675) and water–nitrogen interaction treatments (PERMANOVA, *F* = 0.952, *P* = 0.533) (**Table 1** and **Figure 2A**).

**Table 1:** Effects of water addition (W), nitrogen addition (N), and their interaction (W × N) on the compositions of soil microbial communities, as determined by PERMANOVA.

		W	N	W × N
Bacteria	*F*	1.800	0.826	0.952
	*P*	**0.005**	0.657	0.533
Fungi	*F*	1.063	2.268	0.894
	*P*	0.386	**0.019**	0.603

*P-values reflecting statistical significance are shown in boldface.*

Both water and nitrogen addition had no influences on fungal species richness and SW index (**Figure 1B** and Supplementary Table S1). Soil fungal community composition was significantly altered under nitrogen treatments (PERMANOVA, *F* = 2.268, *P* = 0.019), but not under water treatments (PERMANOVA, *F* = 1.063, *P* = 0.386) and water–nitrogen interaction treatments (PERMANOVA, *F* = 0.894, *P* = 0.603) (**Table 1** and **Figure 2B**).

### Relative Abundance of Dominant Microbial Taxa

**FIGURE 3:**
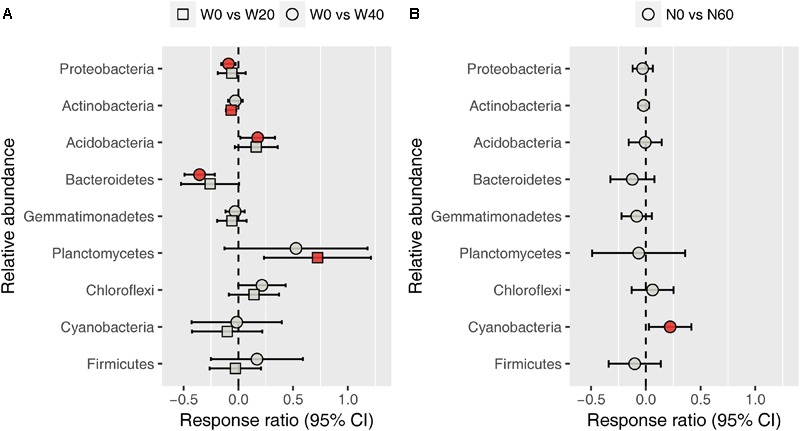
Response ratio analysis of changes in the relative abundance of dominant bacterial phyla in response to water treatment **(A)** and nitrogen treatment **(B)** compared to the control treatment, at the 95% confidence interval. Red points indicate significant changes compared with the control treatment.

Soil bacterial community was dominated by *Proteobacteria* (38.11%), *Actinobacteria* (31.09%), *Acidobacteria* (5.46%), *Bacteroidetes* (4.79%), *Gemmatimonadetes* (4.52%), *Planctomycetes* (4.49%), *Chloroflexi* (4.07%), *Cyanobacteria* (2.40%), and *Firmicutes* (1.04%), based on the analysis of all soil samples (Supplementary Figure S2). Water addition had significant impacts on the relative abundance of *Bacteroidetes*, whereas nitrogen addition, alone or in combination with water, had no effects on the dominant bacterial phyla (Supplementary Table S2). Watering reduced the relative abundance of *Bacteroidetes* from 5.72 ± 0.59 (W0) to 4.61 ± 0.48 (W20) and 4.06 ± 0.24% (W40). Response ratio analysis showed that the relative abundances of *Proteobacteria, Actinobacteria*, and *Bacteroidetes* decreased with water addition, while *Acidobacteria* and *Planctomycetes* exhibited the opposite trend (**Figure 3A**). Nitrogen enrichment increased the response ratio of the *Cyanobacteria* relative abundance, but had no effects on that of other dominant bacterial phyla (**Figure 3B**).

**FIGURE 4:**
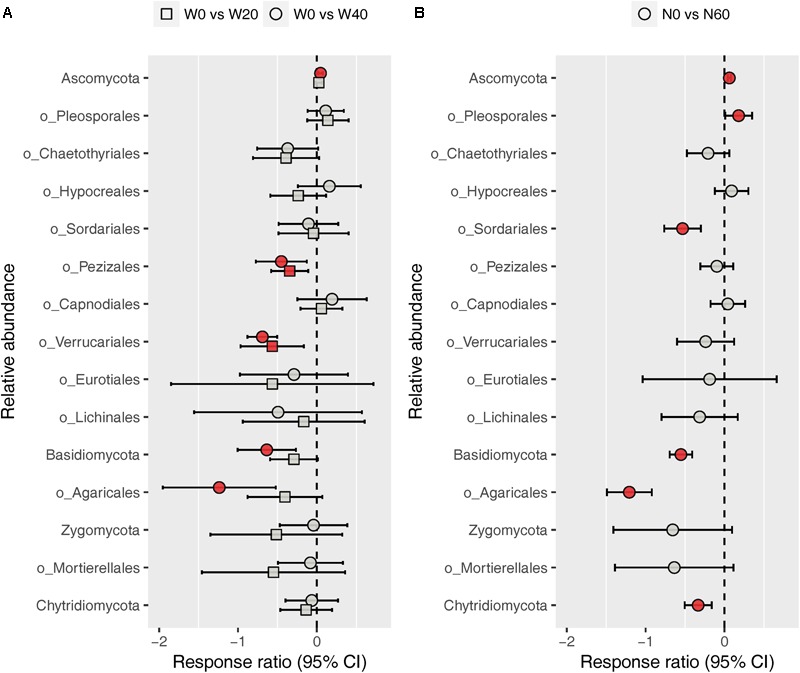
Response ratio analysis of changes in the relative abundance of dominant fungal phyla/orders in response to water treatment **(A)** and nitrogen treatment **(B)** compared to the control treatment, at the 95% confidence interval. Red points indicate significant changes compared with the control treatment.

The fungal community was dominated by *Ascomycota* (88.03%) and *Basidiomycota* (8.32%), while *Zygomycota* (1.59%) and *Chytridiomycota* (1.03%) were minor phyla under all treatments (Supplementary Figure S3). At the order level, we identified nine dominant orders (*Pleosporales*, 38.21%; *Chaetothyriales*, 15.63%; *Hypocreales*, 6.68%; *Sordariales*, 4.13%; *Pezizales*, 2.86%; *Capnodiales*, 2.72%; *Verrucariales*, 1.88%; *Eurotiales*, 1.80%; and *Lichinales*, 1.18%) in the *Ascomycota* division, and one order each in the *Basidiomycota* (*Agaricales*, 5.03%) and *Zygomycota* (*Mortierellales*, 1.57%) division among all soil samples studied (Supplementary Figure S4). ANOVA results showed that nitrogen enrichment significantly influenced the relative abundances of the *Ascomycota* and *Basidiomycota* phyla and the *Pleosporales, Sordariales*, and *Agaricales* orders, whereas water addition and water–nitrogen interactions showed no effects on the relative abundances of any dominant fungal taxa (Supplementary Table S3). Relative to the unfertilized plots, nitrogen addition increased the relative abundances of *Ascomycota* and *Pleosporales* by 6.38 and 23.53%, respectively, but reduced those of *Basidiomycota, Sordariales*, and *Agaricales* by 41.12, 37.53, and 67.73%, respectively. Response ratio analysis showed similar results to ANOVA, in terms of the phylum- and order-level responses of fungi to nitrogen addition (**Figure 4B**). In addition, response ratio analysis also demonstrated that water addition had remarkable impacts on dominant fungal taxa, with *Ascomycota* increasing but *Basidiomycota, Agaricales, Pezizales*, and *Verrucariales* decreasing in their relative abundances (**Figure 4A**).

### Relationships between Microbial Communities and Plant/Soil Properties

**FIGURE 5:**
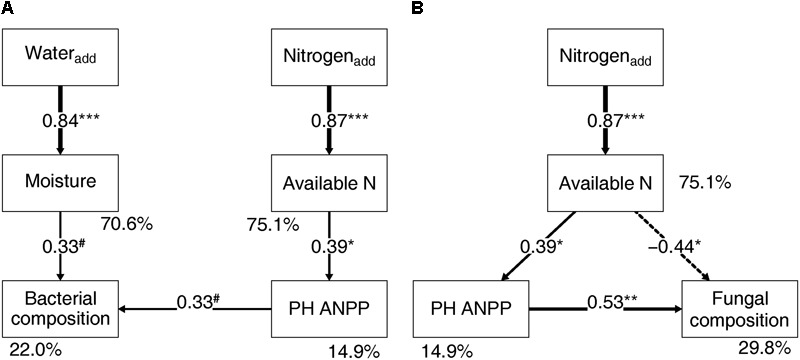
Structural equation modeling showing the relationships between plant/soil properties and the bacterial **(A)** and fungal **(B)** community compositions. Solid arrows indicate positive effects, and the dashed arrow indicates a negative correlation. The standardized path coefficients are adjacent to the arrows and indicate the effect size of the relationship. Arrow widths are proportional to the strength of each relationship. Percentages beside the response variables refer to the proportion of variance explained by the model (*R*^2^). Results of model fitting: **(A)** bacteria: χ^2^ = 10.096, *df* = 9, *P* = 0.343; CFI = 0.984; AIC = 353.187; RMSEA = 0.071, *P* = 0.391; **(B)** fungi: χ^2^ = 2.052, *df* = 2, *P* = 0.358; CFI = 0.999; AIC = 238.585; RMSEA = 0.033, *P* = 0.380. PH ANPP, the aboveground net primary productivity of perennial herbs. ^#^*P* < 0.07, ^∗^*P* < 0.05, ^∗∗^*P* < 0.01, and ^∗∗∗^*P* < 0.001.

Soil bacterial community composition strongly correlated with PH ANPP, STN, and STP, but weakly correlated with soil moisture (**Table 2**). Stepwise regression analysis demonstrated that bacterial diversity and the relative abundances of *Bacteroidetes* and *Planctomycetes*, whose value being altered by water addition, were mainly affected by soil moisture and total nitrogen content; those of *Proteobacteria* and *Acidobacteria* were shifted by watering, but were unrelated to any plant and soil properties measured in our study; and that of *Cyanobacteria*, whose abundance being increased by nitrogen addition, showed positive correlations with the ANPP of perennial shrub and herbs (**Table 3**). SEM results showed that water addition directly affected soil bacterial community by increasing soil moisture, while nitrogen enrichment indirectly influenced the bacterial community by changing PH ANPP (**Figure 5A**). The total net effect of water addition on the bacterial community was marginally significant [standardized coefficient = 0.28, *Z*_value_ (dividing the regression weight estimate by its standard error) = 1.79, *P* = 0.073], whereas that of nitrogen addition was non-significant (standardized coefficient = 0.11, *Z*_value_ = 1.35, *P* = 0.176).

**Table 2:** Correlations between plant/soil properties and soil microbial communities (Bray–Curtis dissimilarities), as determined by Mantel test.

Properties	Bacteria		Fungi	
	*r*	*P*	*r*	*P*
Plant SW	-0.103	0.820	-0.076	0.778
Shrub ANPP	-0.050	0.652	0.154	0.094
PH ANPP	0.264	**0.032**	0.111	0.166
AS ANPP	-0.112	0.790	0.182	0.064
Moisture	0.128	0.092	0.055	0.268
DIN	-0.057	0.711	0.112	0.101
Soil pH	-0.045	0.683	-0.017	0.577
SOC	0.113	0.135	0.075	0.190
STN	0.313	**0.010**	0.183	**0.040**
STP	0.391	**0.004**	0.082	0.215

*P-values reflecting statistical significance are shown in boldface.*

*ANPP, aboveground net primary productivity; AS, annuals; DIN, dissolvable inorganic nitrogen; PH, perennial herbs; SOC, soil organic carbon; STN, soil total nitrogen; STP, soil total phosphorous; SW, Shannon–Wiener index.*

**Table 3:** Results of stepwise regression analysis of the relationships between plant/soil properties and the diversity and relative abundance of various microbial groups.

Diversity/relative abundance	Model	*R^2^*	*F*	*P*
**Bacterial SR**	*y* = 4101.935 + 104.470 (moisture) - 2591.638 (STN)	0.383	6.520	0.006
**Bacterial SW**	*y* = 9.795 + 0.081 (moisture) - 1.858 (STN)	0.459	8.924	0.002
**Proteobacteria**	–	0.361	12.452	0.002
Actinobacteria	*y* = 0.376 - 0.235 (STN)			
**Acidobacteria**	–	0.444	8.389	0.002
**Bacteroidetes**	*y* = 0.038 - 0.005 (moisture) + 0.151 (STN)	0.463	4.098	0.015
Gemmatimonadetes	*y*= 0.098 - 0.006 (shrub ANPP) - 0.004 (PH ANPP) + 0.001 (AS ANPP) - 0.046 (STN)	0.130	3.279	0.084
**Planctomycetes**	*y* = 0.007 + 0.006 (moisture)	0.331	5.187	0.015
Chloroflexi	*y* = 0.053 + 0.003 (moisture) - 0.107 (STN)	0.361	12.452	0.002
**Cyanobacteria**	*y* = -0.023 + 0.007 (shrub ANPP) + 0.004 (PH ANPP)	0.311	4.746	0.020
Firmicutes	*y* = 0.018 - 0.026 (STN)	0.134	3.415	0.078

Fungal SR	–			
Fungal SW	*y* = 6.684 - 0.370 (shrub ANPP)	0.173	4.589	0.043
**Ascomycota**	*y* = 0.556 + 0.045 (shrub ANPP) + 0.011 (moisture) + 0.023 (DIN)	0.562	8.545	0.001
**o_Pleosporales**	*y* = 0.272 + 0.002 (AS ANPP) + 0.043 (DIN)	0.550	12.811	0.000
o_Chaetothyriales	*y* = -3.452 + 0.340 (shrub ANPP) - 0.011 (AS ANPP)	0.262	3.733	0.041
o_Hypocreales	*y* = 0.095 + 0.011 (PH ANPP) - 0.222 (STN)	0.351	5.683	0.011
**o_Sordariales**	*y* = 0.063 + 2.928^∗^10^-4^ (AS ANPP) - 0.010 (DIN)	0.391	6.754	0.005
**o_Pezizales**	*y* = 0.045 - 0.003 (moisture)	0.206	5.695	0.026
o_Capnodiales	*y* = -2.367 - 0.328 (shrub ANPP) + 0.168 (DIN)	0.288	4.248	0.028
**o_Verrucariales**	*y* = 0.012 - 0.004 (moisture) + 0.117 (STN)	0.258	3.659	0.043
o_Eurotiales	*y* = 0.033 + 0.004 (PH ANPP) - 0.097 (STN)	0.368	6.116	0.008
o_Lichinales	–			
**Basidiomycota**	*y* = 0.368 - 0.462 (shrub ANPP) - 0.123 (moisture)	0.510	10.942	0.001
**o_Agaricales**	*y* = 3.567 - 1.167 (shrub ANPP) - 0.405 (PH ANPP)	0.524	11.561	<0.001
Zygomycota	–			
o_Mortierellales	–			
**Chytridiomycota**	*y* = - 4.349 - 0.174 (DIN)	0.169	4.487	0.046

*Groups in boldface type are those that showed significant responses to water and/or nitrogen addition, as assessed by two-way ANOVA and response ratio analysis.*

*ANPP, aboveground net primary productivity; AS, annuals; DIN, dissolvable inorganic nitrogen; PH, perennial herbs; STN, soil total nitrogen; SR, species richness; SW, Shannon–Wiener index.*

The fungal community composition strongly correlated with STN and weakly correlated with the ANPP of shrub and annual plants (**Table 2**). Stepwise regression analysis revealed that fungal diversity and the relative abundances of *Ascomycota* and *Basidiomycota* strongly correlated with shrub ANPP (**Table 3**). The dominant fungal phyla *Ascomycota, Basidiomycota*, and *Chytridiomycota* were shifted by water and nitrogen addition, and also showed strong correlations with soil moisture and/or available nitrogen (**Table 3**). In the order level, the relative abundances of *Pezizales* and *Verrucariales*, whose abundance being reduced by water addition, were negatively correlated with soil moisture and total nitrogen content; that of *Pleosporales* increased with nitrogen enrichment and showed positive correlations with AS ANPP and soil available nitrogen; that of *Sordariales*, whose abundance being reduced by nitrogen fertilization, showed a positive correlation with AS ANPP, but a negative correlation with soil available nitrogen; that of *Agaricales*, whose abundance being decreased in both water and nitrogen treatments, showed negative correlations with the ANPP of perennial shrub and herbs (**Table 3**). SEM analysis demonstrated that nitrogen enrichment could directly affect the fungal community by increasing nitrogen availability and indirectly via changing PH ANPP (**Figure 5B**). The total net direct effect of nitrogen enrichment was significant (standardized coefficient = -0.38, *Z*_value_ = -2.30, *P* = 0.022), whereas the indirect effect was non-significant (standardized coefficient = 0.18, *Z*_value_ = 1.64, *P* = 0.101).

## Discussion

### Precipitation Increment But Not Nitrogen Enrichment Significantly Influenced Desert Bacterial Community

Our results showed that water addition significantly increased soil bacterial diversity, and altered the bacterial community composition, with *Acidobacteria* and *Planctomycetes* increasing but *Proteobacteria* and *Bacteroidetes* decreasing in terms of their relative abundances (**Figures 1–3** and **Table 1**). These results suggest that water addition tended to promote flourishing of oligotrophic taxa and depress copiotrophic taxa. Many species of oligotrophic taxa (e.g., *Acidobacteria*) have been shown to act as stress tolerators, which can enter a dormant state to evade stressful conditions (Fierer, 2017; Tecon and Or, 2017). As desert environments are water-stressful, alleviating that stress following water addition would revive dormant microorganisms and increase their abundances (Tecon and Or, 2017). However, this shifting trend in bacterial taxa in our study contrasted with the results from studies performed in a temperate grassland, in which water addition tends to increase the relative abundance of *Proteobacteria* and decrease that of *Acidobacteria* (Zhang et al., 2013, 2014). One possible mechanism underlying the responses of bacterial phyla to water addition in grasslands was that watering could increase nitrogen availability by stimulating the mineralization of soil organic matter (Li et al., 2016, 2017b). However, in our study, soil nitrogen availability was not affected by water addition, probably due to the quite low amount of soil organic matter (Supplementary Table S1), which is roughly equivalent to one-seventh of that in grassland soils (Zeng et al., 2016; Li et al., 2017b). Alternatively, this contrary response was likely due to the high sensitivity of oligotrophic taxa to water addition in the more stressful desert environment. Evidence from aridity-gradient studies in northern China indicates that the relative abundances of *Acidobacteria* and *Planctomycetes* increase with an increasing aridity index (AI, estimated by the ratio of precipitation to potential evapotranspiration) in more arid areas (AI < 0.2), but exhibit no further variation at higher AI values (Wang X. et al., 2015; Wang et al., 2017); whereas the relative abundances of *Bacteroidetes* and most *Proteobacteria* subphyla show non-linear relationships with AI, with the lowest value being present at AI ≈ 0.2 (Wang X. et al., 2015). The AI in our study site is 0.14; thus, oligotrophic taxa were expected to increase and copiotrophic taxa were expected to decrease with water addition. The dormancy of oligotrophic taxa might be more prevalent in more stressful environments, resulting in a larger population recovery when conditions improve and further depress copiotrophic taxa.

In contrast to previous findings, neither the bacterial diversity nor the community composition was substantially affected by nitrogen addition in our study (**Figures 1, 2** and **Table 1**). Among all dominant bacterial phyla identified, only *Cyanobacteria* were slightly increased with nitrogen input in terms of the relative abundance (**Figure 3B**). Previously, the relative abundance of *Cyanobacteria* has been shown a non-linear response to nitrogen enrichment, with an increase under moderate nitrogen input (35-70 kg N ha^-1^ yr^-1^) and a decrease under excess nitrogen addition (140 kg N ha^-1^ yr^-1^) (Wang J. et al., 2015). The overall unresponsiveness of the bacterial community following nitrogen enrichment was also reported in fertilization experiments conducted in semiarid grasslands (Carey et al., 2015; McHugh et al., 2017). There are several potential explanations regarding these insensitive responses. The low nitrogen-retention capacity of desert soil is likely to weaken the effects of nitrogen addition (Jin et al., 2015; McHugh et al., 2017). In addition, data from numerous fertilization studies have shown that nitrogen-induced soil acidification is an important mechanism in shifting the bacterial community composition (Chen et al., 2015; Zeng et al., 2016; Zhang et al., 2017b). However, in our study, nitrogen addition did not influence the soil pH (Supplementary Table S1), which was likely due to the high buffering capacity of the soil (Liu et al., 2015). Although direct effects of nitrogen addition on the bacterial community were not detected, our results suggest that nitrogen addition could indirectly influence the bacterial community by changing PH ANPP (**Figure 5A**). In grasslands, many field studies have shown that nitrogen-induced changes in aboveground plant biomass are important drivers of shifting bacterial communities (Chen et al., 2015; Yuan et al., 2016). We also found that nitrogen addition increased the biomass of PH (Supplementary Table S1), although this indirect effect was weak (**Figure 5A**). It is possible that the effects of nitrogen deposition, as seen in the fertilization experiments, take longer to emerge than the 2-year observation period of this study.

### Increased Precipitation and Nitrogen Altered the Drought-Tolerant and Trophic Traits of Desert Fungal Community

In contrast to the bacterial community, both water and nitrogen addition significantly impacted the fungal community composition, but not the fungal diversity (**Figures 1, 2** and **Table 1**). Nitrogen addition mainly increased the relative abundance of *Ascomycota* and decreased that of *Basidiomycota* (**Figure 4B** and Supplementary Table S3). It was suggested that the phyla *Ascomycota* and *Basidiomycota* could roughly grouped into copiotrophic and oligotrophic taxa, respectively (Ho et al., 2017; Yao et al., 2017), suggesting that they would respond oppositely to nitrogen enrichment. Moreover, many field experiments conducted in diverse ecosystems have also revealed that *Basidiomycota* decreased in relative abundance following nitrogen input (Allison et al., 2007; Entwistle et al., 2013; Chen et al., 2018). We also found that the relative abundance of *Chytridiomycota* decreased under nitrogen treatment; however, its relative proportion was quite small (∼1%), and thus its variation can be negligible. The increased relative abundance of *Ascomycota* following nitrogen addition was primarily due to an increased relative abundance of *Pleosporales*, which was the predominant fungal order in our study. Members in the order *Pleosporales* have been shown to live as plant endophytes in arid grass species and were shown to transfer nutrients between plants and nearby soil (Green et al., 2008; Porras-Alfaro et al., 2011). Our stepwise regression analysis also indicated that the relative abundance of *Pleosporales* was positively correlated with annual plants and soil inorganic nitrogen (**Table 3**). Previous data also demonstrated that the relative abundance of *Pleosporales* increases with nitrogen enrichment (Lowell and Klein, 2001; Wang J. et al., 2015), which supports our findings. In contrast to *Pleosporales*, members of the orders *Sordariales* (*Ascomycota*) and *Agaricales* (*Basidiomycota*) are considered as the potent degraders of lignin and predominantly show oligotrophic features (Poggeler, 2011; Entwistle et al., 2013; Ho et al., 2017). Results from stepwise regression showed that the relative abundance of *Sordariales* was negatively correlated with soil available nitrogen, while that of *Agaricales* showed a negative correlation with PH ANPP, suggesting that nitrogen enrichment was likely to directly affect *Sordariales* relative abundance via increasing nitrogen availability, whereas indirectly influence *Agaricales* by changing PH ANPP (**Table 3**). Taken together, our results indicated that nitrogen enrichment tended to depress oligotrophic fungal taxa, but to facilitate taxa regarding nutrient transfer.

Our response ratio analysis indicated that water addition tended to favor *Ascomycota* over *Basidiomycota* (**Figure 4A**). The ANOVA and PERMANOVA results showed that the fungal community composition was unresponsive to water addition (**Table 1**, **Figure 2**, and Supplementary Table S3), probably due to the large variation in our fungal data. Data from many field experiments have demonstrated that drought treatment decreases the relative abundance of *Ascomycota* and increases that of *Basidiomycota* (McHugh and Schwartz, 2014; He et al., 2016; Bastida et al., 2017), suggesting that *Basidiomycota* adapt better to drought conditions. Our stepwise regression analysis also revealed that the relative abundance of *Ascomycota* was positively correlated with soil moisture, while that of *Basidiomycota* exhibited an opposite pattern (**Table 3**). Analyzing the order-level responses of fungi to water addition demonstrated that the relative abundances of *Pezizales* (*Ascomycota*), *Verrucariales* (*Ascomycota*), and *Agaricales* (*Basidiomycota*) decreased with water input, indicating that the change of *Basidiomycota* was mainly caused by *Agaricales* and the increase in *Ascomycota* relative abundance was likely driven by many low-abundance taxa. Results from stepwise regression showed that the relative abundances of *Pezizales* and *Verrucariales* were negatively correlated with soil moisture (**Table 3**), suggesting that these fungal orders might prefer to live in drought conditions. Previous studies have also revealed that species of the order *Pezizales* are important members of ectomycorrhizal fungi (Tedersoo et al., 2006; Healy et al., 2013) and adapt well in water-stressed environments (Smith et al., 2006; Gordon and Gehring, 2011). In contrast to *Pezizales*, most species of the order *Verrucariales* are characterized as lichen-forming fungi (Gueidan et al., 2007; Wang et al., 2014). Previous physiological data indicated that, in arid areas, these fungi are extremely drought-tolerant and, thus, are expected to adapt well in desert environments (Wang Y. et al., 2015; Zhang et al., 2017a). These studies indirectly supported our findings that water addition reduced the relative abundances of drought-adapted fungi.

### Precipitation and Nitrogen Addition Primarily Directly Affected Desert Microbial Communities via Changing Resource Availability

The present study indicated that water and nitrogen addition resulted in stronger direct effects on soil microbial communities through changing resource availability rather than indirect influences via changes of plant community (**Table 3** and **Figure 5**). Specially, shifts in the fungal community composition following water and nitrogen enrichment were primarily caused by the changes of water and nitrogen availability, whereas shifts in the bacterial community composition were mainly driven by changes in soil moisture. Fungal and bacterial communities responded in different ways to resource availability, probably due to their distinctive adaptive strategies to desert environments. Fungi are typically more drought-tolerant and nutrient-sensitive than bacteria, attributed to their ability to access soil water and nutrients better through hyphal networks (Boer et al., 2005; Yuste et al., 2011; Manzoni et al., 2012). Consequently, when water and nitrogen availability are improved, drought- and oligotrophic-adapted fungal taxa are expected to be suppressed (Crowther et al., 2014). However, desert bacteria probably show limited responses to a moderate rate of nitrogen fertilization due to their relatively low ability for nutrient acquisition and the low nutrient-retention capacity of desert soils. In contrast to nitrogen availability, desert bacteria are likely more sensitive to the improvement of soil moisture, probably due to the revival of dormant taxa when water stress is alleviated (Lennon and Jones, 2011; Tecon and Or, 2017).

The SEM results suggest that nitrogen enrichment could indirectly influence soil microbial communities via altering PH ANPP, while the indirect effects of water addition were not detectable (**Figure 5**). Although the indirect nitrogen effects were found in our study, their strength was relatively weaker than the direct effects. Our findings were not in line with the results reported in grassland ecosystems, where nitrogen-induced shifts in microbial community composition are mainly indirectly mediated by changes of soil pH and/or plant community rather than directly via changing resource availability (Chen et al., 2015; Yuan et al., 2016; Zeng et al., 2016). It is likely that desert ecosystems are more resource limited than grasslands, suggesting that desert microbial communities are likely more sensitive to resource availability. The high buffering capacity of our soils might weaken the effects of nitrogen-induced soil acidification. It is also possible that a stronger indirect effect of nitrogen enrichment can be seen in a long-term experimental treatment. Owing to limitations of our short-term experiment, further filed studies with longer observation period will be necessary to disentangle the direct and indirect influences of global environmental changes on desert microbial communities.

## Conclusions

In summary, our results indicated that soil microbial communities responded differently to increased precipitation and nitrogen in this desert ecosystem. Watering increased soil bacterial diversity and shifted the community composition by promoting the flourishing of stress-tolerant (dormant) taxa, whereas nitrogen enrichment had no substantial effects. Water and nitrogen addition did not influence soil fungal diversity, but significantly altered the community composition with drought-adapted taxa being suppressed following water addition and with oligotrophic-adapted taxa being suppressed under nitrogen enrichment. Water- and nitrogen-induced changes in soil microbial communities arose mainly through altering resource availability rather than plant community. Our results suggest that water addition affected desert microbial communities by altering their stress-tolerant traits, while nitrogen enrichment shifted their copiotrophic/oligotrophic traits. Although, in a short-term experiment, our findings highlight the importance of resource availability in driving the desert microbial responses to altered environmental conditions, further long-term study is needed to help in better understanding the responses of desert microbial communities to global environmental changes.

## Author Contributions

WS, YZ, and SQ conceived of the study and designed the methodology. WS, YB, and JZ collected the data. WS analyzed the data. WS led the writing of the manuscript. YB, YZ, SQ, WF, YS, and BW assisted with revising the draft manuscript. All authors approved of the final manuscript.

## Conflict of Interest Statement

The authors declare that the research was conducted in the absence of any commercial or financial relationships that could be construed as a potential conflict of interest.
