# Supplementary material for: Resource Availability Drives Responses of Soil Microbial Communities to Short-term Precipitation and Nitrogen Addition in a Desert Shrubland
Source: Front Microbiol. 2018 Feb 9;9:186. doi: 10.3389/fmicb.2018.00186 (PMC5811472; doi:10.3389/fmicb.2018.00186)

**Supplementary Material**

**Resource availability drives responses of soil microbial communities to short-term precipitation and nitrogen addition in a desert shrubland**

Weiwei She, Yuxuan Bai, Yuqing Zhang^*^, Shugao Qin, Wei Feng, Yanfei Sun, Jing Zheng, Bin Wu

**Table S1.** Results of two-way ANOVA of the effects of water addition (W), nitrogen addition (N), and their interaction (W × N) on plant, soil, and microbial properties. The mean value and standard error (n = 4) of each property at each water-treatment level (W0, W20, and W40) and each nitrogen-addition rate (N0 and N60) are shown in the right columns of the table. *P* values reflecting statistical significance are shown in boldface. Lowercase letters in the right columns of the table indicate significant differences observed following water or nitrogen addition (*P* < 0.05, one-way ANOVA). Abbreviations: ANPP, aboveground net primary productivity; AS, annuals; DIN, dissolvable inorganic nitrogen; PH, perennial herbs; SOC, soil organic carbon; STN, soil total nitrogen; STP, soil total phosphorous; SR, species richness; SW, Shannon-Wiener index

| Properties | | W | N | W × N |  |  | W0 | W20 | W40 | N0 | N60 |
| --- | --- | --- | --- | --- | --- | --- | --- | --- | --- | --- | --- |
| Plant SW | *F* | 7.180 | 0.093 | 1.185 |  | *mean* | **1.14 b** | **1.23 b** | **1.40 a** | 1.27 | 1.25 |
|  | *P* | **0.006** | 0.765 | 0.333 |  | *se* | 0.05 | 0.04 | 0.05 | 0.06 | 0.04 |
| Shrub ANPP (g m^−2^) | *F* | 0.116 | 0.055 | 3.390 |  | *mean* | 146.13 | 149.73 | 161.26 | 149.24 | 155.50 |
|  | *P* | 0.891 | 0.818 | 0.061 |  | *se* | 24.05 | 22.26 | 39.31 | 29.58 | 15.55 |
| PH ANPP (g m^−2^) | *F* | 2.169 | 7.550 | 0.815 |  | *mean* | 23.67 | 36.93 | 57.94 | **20.90 b** | **58.13 a** |
|  | *P* | 0.149 | **0.015** | 0.461 |  | *se* | 7.30 | 16.44 | 18.30 | 6.15 | 14.66 |
| AS ANPP (g m^−2^) | *F* | 1.924 | 2.422 | 0.952 |  | *mean* | 7.26 | 25.69 | 12.82 | 9.13 | 21.38 |
|  | *P* | 0.180 | 0.140 | 0.408 |  | *se* | 3.18 | 12.29 | 4.68 | 3.90 | 8.16 |
| Moisture (%) | *F* | 25.772 | 0.139 | 1.190 |  | *mean* | **4.18 c** | **6.04 b** | **7.53 a** | 5.99 | 5.85 |
|  | *P* | **<0.001** | 0.715 | 0.331 |  | *se* | 0.27 | 0.38 | 0.36 | 0.55 | 0.43 |
| DIN (mg N kg^−1^) | *F* | 0.413 | 49.334 | 1.273 |  | *mean* | 1.63 | 1.62 | 1.82 | **0.98 b** | **2.40 a** |
|  | *P* | 0.669 | <**0.001** | 0.309 |  | *se* | 0.25 | 0.24 | 0.42 | 0.08 | 0.17 |
| Soil pH | *F* | 1.235 | 0.058 | 0.970 |  | *mean* | 8.13 | 8.13 | 8.09 | 8.12 | 8.11 |
|  | *P* | 0.319 | 0.813 | 0.402 |  | *se* | 0.03 | 0.02 | 0.02 | 0.02 | 0.02 |
| SOC (g kg^−1^) | *F* | 0.496 | 0.887 | 1.768 |  | *mean* | 2.72 | 3.08 | 2.83 | 3.02 | 2.73 |
|  | *P* | 0.619 | 0.361 | 0.204 |  | *se* | 0.30 | 0.41 | 0.23 | 0.28 | 0.24 |
| STN (g kg^−1^) | *F* | 0.383 | 0.859 | 1.071 |  | *mean* | 0.26 | 0.29 | 0.28 | 0.29 | 0.26 |
|  | *P* | 0.688 | 0.369 | 0.367 |  | *se* | 0.03 | 0.03 | 0.01 | 0.02 | 0.02 |
| STP (g kg^−1^) | *F* | 0.059 | 0.043 | 3.571 |  | *mean* | 0.24 | 0.25 | 0.24 | 0.24 | 0.24 |
|  | *P* | 0.943 | 0.838 | 0.054 |  | *se* | 0.02 | 0.02 | 0.02 | 0.02 | 0.02 |
| Bacterial SR | *F* | 4.949 | 0.337 | 1.963 |  | *mean* | **3848.6 b** | **4013.4 ab** | **4153.1 a** | 3982.1 | 4028.0 |
|  | *P* | **0.022** | 0.570 | 0.175 |  | *se* | 114.5 | 102.7 | 47.6 | 98.1 | 64.0 |
| Bacterial SW | *F* | 8.746 | 0.327 | 0.743 |  | *mean* | **9.64 b** | **9.77 a** | **9.88 a** | 9.75 | 9.77 |
|  | *P* | **0.003** | 0.576 | 0.492 |  | *se* | 0.08 | 0.07 | 0.03 | 0.06 | 0.06 |
| Fungal SR | *F* | 0.833 | 0.001 | 0.684 |  | *mean* | 159.6 | 170.3 | 171.5 | 167.3 | 167.0 |
|  | *P* | 0.454 | 0.976 | 0.519 |  | *se* | 11.2 | 10.0 | 3.5 | 8.0 | 6.4 |
| Fungal SW | *F* | 0.015 | 0.246 | 1.041 |  | *mean* | 4.89 | 4.88 | 4.85 | 4.91 | 4.83 |
|  | *P* | 0.985 | 0.627 | 0.377 |  | *se* | 0.14 | 0.22 | 0.17 | 0.15 | 0.13 |

**Table S2.** Results of two-way ANOVA of the effects of water addition (W), nitrogen addition (N), and their interaction (W × N) on the relative abundances of soil bacterial phyla (≥1%). The mean value and standard error (n = 4) of each taxon at each water-treatment level (W0, W20, and W40) and each nitrogen-addition rate (N0 and N60) are shown in the right columns of the table. *P* values reflecting statistical significance are shown in boldface. Lowercase letters in the right columns of the table indicate significant differences observed following water or nitrogen addition (*P* < 0.05, one-way ANOVA).

| Phyla |  | W | N | W × N |  |  | W0 | W20 | W40 | N0 | N60 |
| --- | --- | --- | --- | --- | --- | --- | --- | --- | --- | --- | --- |
| Proteobacteria | *F* | 1.590 | 0.248 | 0.239 |  | *mean* | 39.88 | 37.90 | 36.55 | 38.49 | 37.73 |
|  | *P* | 0.237 | 0.626 | 0.790 |  | *se* | 0.99 | 1.88 | 1.03 | 0.63 | 1.51 |
| Actinobacteria | *F* | 1.325 | 0.280 | 0.612 |  | *mean* | 32.04 | 30.02 | 31.23 | 31.36 | 30.82 |
|  | *P* | 0.295 | 0.605 | 0.555 |  | *se* | 1.06 | 0.60 | 0.89 | 0.72 | 0.74 |
| Acidobacteria | *F* | 1.939 | 0.052 | 0.452 |  | *mean* | 4.80 | 5.78 | 5.81 | 5.41 | 5.52 |
|  | *P* | 0.178 | 0.822 | 0.645 |  | *se* | 0.39 | 0.47 | 0.38 | 0.34 | 0.38 |
| Bacteroidetes | *F* | 5.913 | 0.911 | 0.615 |  | *mean* | **5.72 a** | **4.61 b** | **4.06 b** | 4.99 | 4.60 |
|  | *P* | **0.013** | 0.355 | 0.554 |  | *se* | 0.59 | 0.48 | 0.24 | 0.44 | 0.40 |
| Gemmatimonadetes | *F* | 0.180 | 0.932 | 1.885 |  | *mean* | 4.63 | 4.42 | 4.52 | 4.67 | 4.38 |
|  | *P* | 0.837 | 0.350 | 0.186 |  | *se* | 0.39 | 0.25 | 0.16 | 0.20 | 0.24 |
| Planctomycetes | *F* | 2.952 | 0.253 | 0.376 |  | *mean* | 2.45 | 5.70 | 5.32 | 4.19 | 4.79 |
|  | *P* | 0.083 | 0.622 | 0.693 |  | *se* | 0.40 | 0.87 | 1.35 | 0.82 | 0.92 |
| Chloroflexi | *F* | 2.820 | 1.464 | 0.178 |  | *mean* | 3.52 | 4.18 | 4.50 | 3.86 | 4.27 |
|  | *P* | 0.091 | 0.245 | 0.839 |  | *se* | 0.29 | 0.38 | 0.42 | 0.25 | 0.37 |
| Cyanobacteria | *F* | 0.159 | 2.458 | 0.006 |  | *mean* | 2.37 | 2.28 | 2.55 | 2.09 | 2.72 |
|  | *P* | 0.854 | 0.138 | 0.994 |  | *se* | 0.28 | 0.32 | 0.36 | 0.24 | 0.24 |
| Firmicutes | *F* | 1.425 | 0.058 | 2.269 |  | *mean* | 0.93 | 0.94 | 1.23 | 1.06 | 1.02 |
|  | *P* | 0.271 | 0.813 | 0.138 |  | *se* | 0.15 | 0.10 | 0.21 | 0.15 | 0.11 |

**Table S3.** Results of two-way ANOVA of the effects of water addition (W), nitrogen addition (N), and their interaction (W × N) on the relative abundances of soil fungal phyla/orders (≥1%). The mean value and standard error (n = 4) of each taxon at each water-treatment level (W0, W20, and W40) and each nitrogen-addition rate (N0 and N60) are shown in the right columns of the table. *P* values reflecting statistical significance are shown in boldface. Lowercase letters in the right columns of the table indicate significant differences observed following water or nitrogen addition (*P* < 0.05, one-way ANOVA).

| Phyla/orders |  | W | N | W × N |  |  | W0 | W20 | W40 | N0 | N60 |
| --- | --- | --- | --- | --- | --- | --- | --- | --- | --- | --- | --- |
| Ascomycota | *F* | 2.199 | 11.576 | 1.451 |  | *mean* | 85.91 | 88.17 | 90.01 | **85.31 b** | **90.75 a** |
|  | *P* | 0.145 | **0.004** | 0.265 |  | *se* | 2.78 | 1.47 | 1.03 | 1.80 | 0.76 |
| *o_Pleosporales* | *F* | 1.784 | 6.760 | 0.647 |  | *mean* | 34.16 | 40.97 | 39.49 | **34.18 b** | **42.23 a** |
|  | *P* | 0.202 | **0.020** | 0.538 |  | *se* | 1.72 | 4.39 | 3.83 | 2.04 | 3.18 |
| *o_Chaetothyriales* | *F* | 0.824 | 0.399 | 0.438 |  | *mean* | 18.48 | 14.29 | 14.12 | 16.62 | 14.64 |
|  | *P* | 0.457 | 0.537 | 0.653 |  | *se* | 1.93 | 3.44 | 2.57 | 2.60 | 1.79 |
| *o_Hypocreales* | *F* | 2.347 | 0.750 | 0.055 |  | *mean* | 6.36 | 5.43 | 8.26 | 6.21 | 7.15 |
|  | *P* | 0.130 | 0.400 | 0.946 |  | *se* | 1.10 | 0.82 | 1.49 | 1.21 | 0.70 |
| *o_Sordariales* | *F* | 0.126 | 7.364 | 0.296 |  | *mean* | 4.04 | 4.38 | 3.98 | **5.09 a** | **3.18 b** |
|  | *P* | 0.882 | **0.016** | 0.748 |  | *se* | 0.52 | 0.85 | 0.70 | 0.59 | 0.34 |
| *o_Pezizales* | *F* | 3.251 | 0.123 | 0.221 |  | *mean* | 3.56 | 2.62 | 2.41 | 2.93 | 2.79 |
|  | *P* | 0.067 | 0.730 | 0.804 |  | *se* | 0.41 | 0.29 | 0.29 | 0.34 | 0.26 |
| *o_Capnodiales* | *F* | 1.643 | 0.490 | 0.912 |  | *mean* | 2.34 | 2.60 | 3.22 | 2.58 | 2.86 |
|  | *P* | 0.226 | 0.494 | 0.423 |  | *se* | 0.25 | 0.33 | 0.66 | 0.38 | 0.37 |
| *o_Verrucariales* | *F* | 2.124 | 0.004 | 1.110 |  | *mean* | 2.62 | 1.69 | 1.34 | 1.90 | 1.87 |
|  | *P* | 0.154 | 0.948 | 0.355 |  | *se* | 0.77 | 0.40 | 0.11 | 0.27 | 0.55 |
| *o_Eurotiales* | *F* | 0.034 | 1.163 | 0.363 |  | *mean* | 1.83 | 1.86 | 1.72 | 1.56 | 2.04 |
|  | *P* | 0.967 | 0.298 | 0.702 |  | *se* | 0.43 | 0.63 | 0.36 | 0.29 | 0.46 |
| *o_Lichinales* | *F* | 0.128 | 0.242 | 0.624 |  | *mean* | 1.16 | 1.27 | 1.12 | 1.25 | 1.12 |
|  | *P* | 0.881 | 0.63 | 0.549 |  | *se* | 0.24 | 0.27 | 0.29 | 0.23 | 0.19 |
| Basidiomycota | *F* | 1.810 | 5.100 | 1.196 |  | *mean* | 10.52 | 8.34 | 6.08 | **10.47 a** | **6.16 b** |
|  | *P* | 0.198 | **0.039** | 0.330 |  | *se* | 2.83 | 1.09 | 0.98 | 1.97 | 0.40 |
| *o_Agaricales* | *F* | 1.503 | 6.566 | 1.366 |  | *mean* | 6.97 | 5.39 | 2.74 | **7.61 a** | **2.46 b** |
|  | *P* | 0.254 | **0.022** | 0.285 |  | *se* | 2.95 | 1.18 | 0.87 | 1.97 | 0.25 |
| Zygomycota | *F* | 0.271 | 1.110 | 1.233 |  | *mean* | 1.64 | 1.37 | 1.77 | 1.83 | 1.36 |
|  | *P* | 0.766 | 0.309 | 0.319 |  | *se* | 0.54 | 0.38 | 0.33 | 0.39 | 0.27 |
| *o_Mortierellales* | *F* | 0.202 | 0.948 | 1.135 |  | *mean* | 1.64 | 1.37 | 1.69 | 1.78 | 1.35 |
|  | *P* | 0.820 | 0.346 | 0.348 |  | *se* | 0.54 | 0.38 | 0.31 | 0.39 | 0.27 |
| Chytridiomycota | *F* | 0.082 | 3.094 | 0.101 |  | *mean* | 1.05 | 0.98 | 1.06 | 1.18 | 0.88 |
|  | *P* | 0.922 | 0.099 | 0.904 |  | *se* | 0.14 | 0.14 | 0.17 | 0.14 | 0.08 |

**Figure S1.** Priori structural equation models of hypothetical relationships between plant/soil variables and bacterial/fungal community composition. Abbreviations: ANPP, aboveground net primary productivity; AS, annuals; PH, perennial herbs


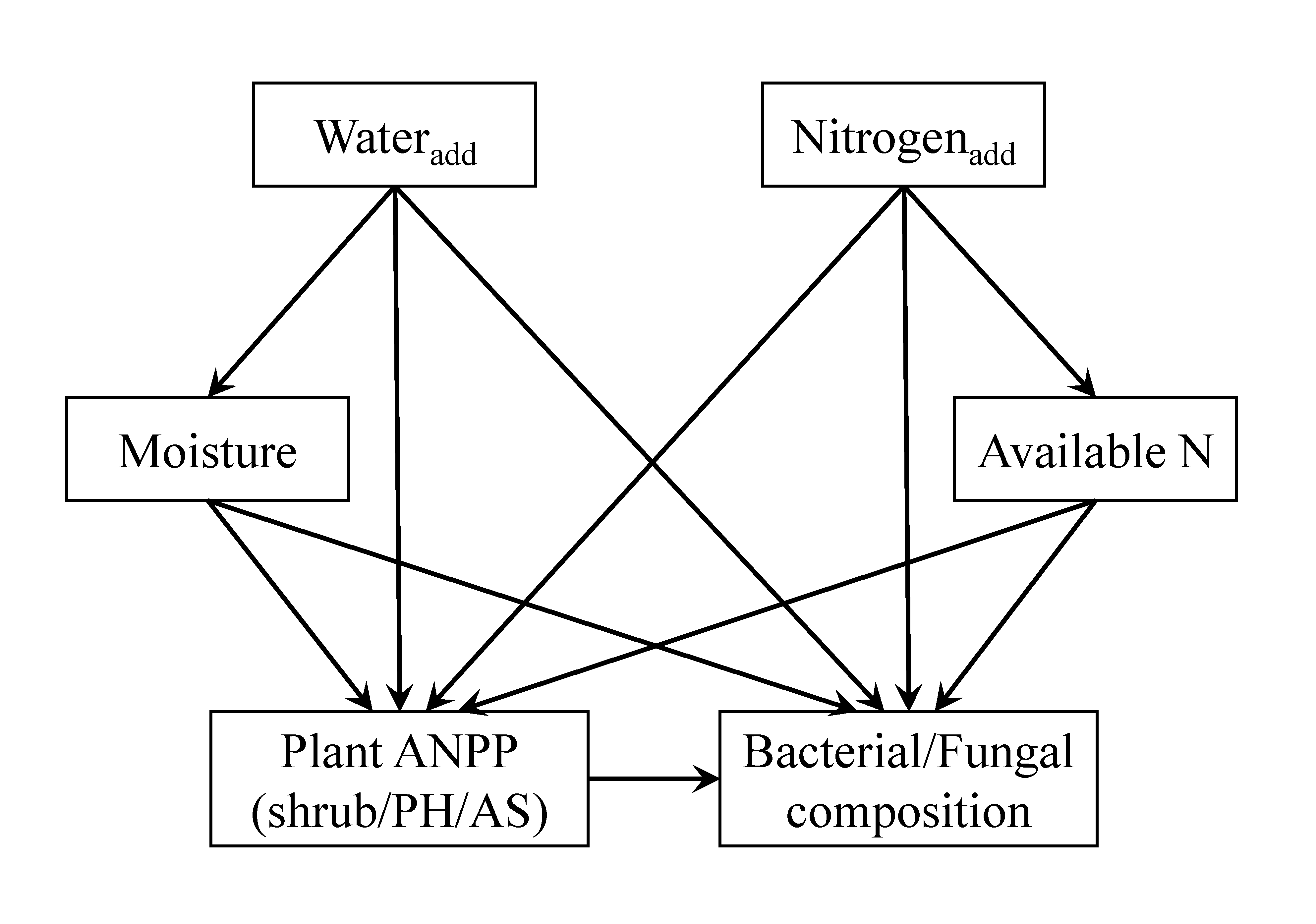


**Figure S2.** Relative abundance of soil bacterial phyla under different water and nitrogen treatments. Individual phyla with a low relative abundance (<1%) were integrated into the “Others” category.


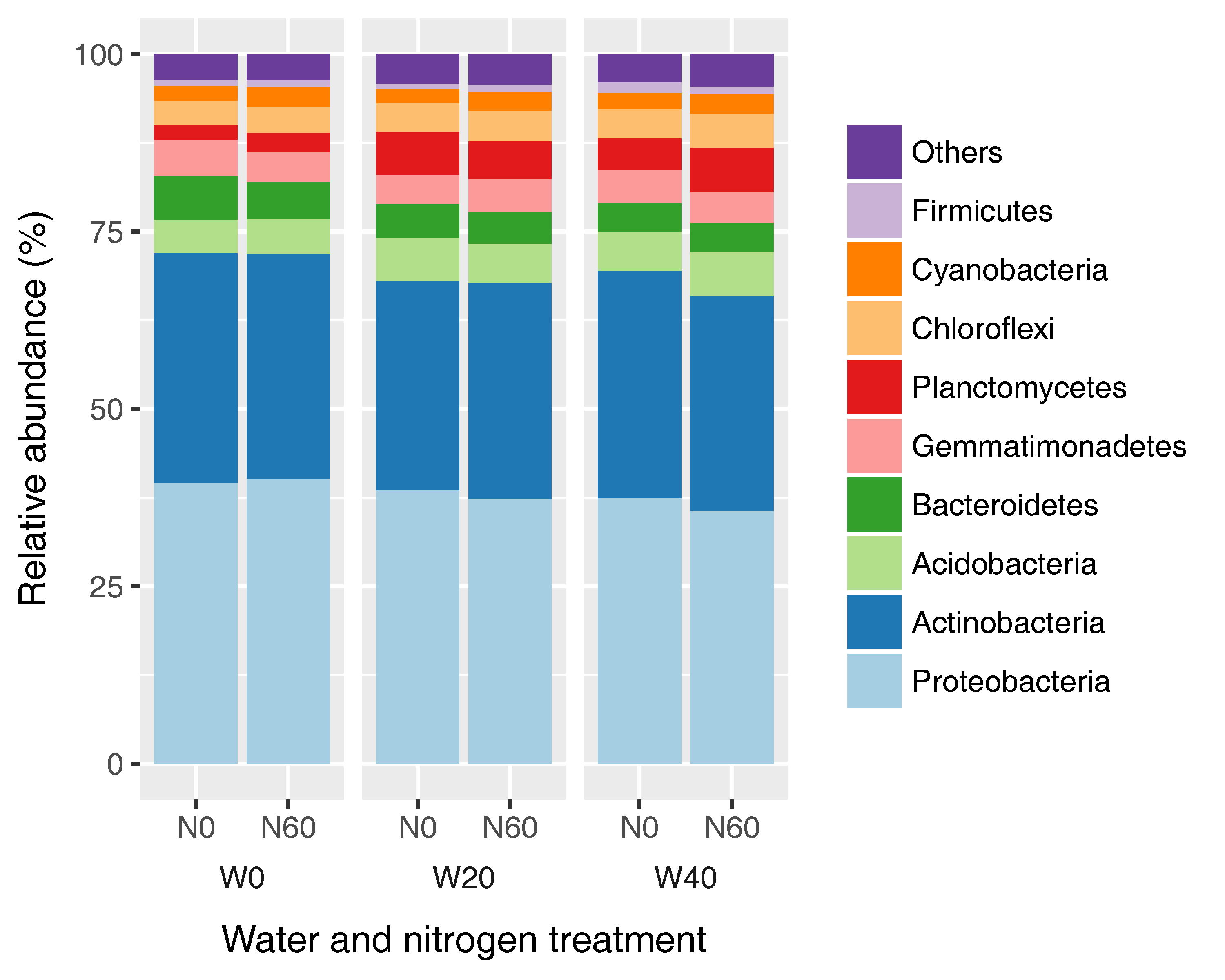


**Figure S3.** Relative abundance of soil fungal phyla under different water and nitrogen treatments. Individual phyla with a low relative abundance (<1%) were integrated into the “Others” category.


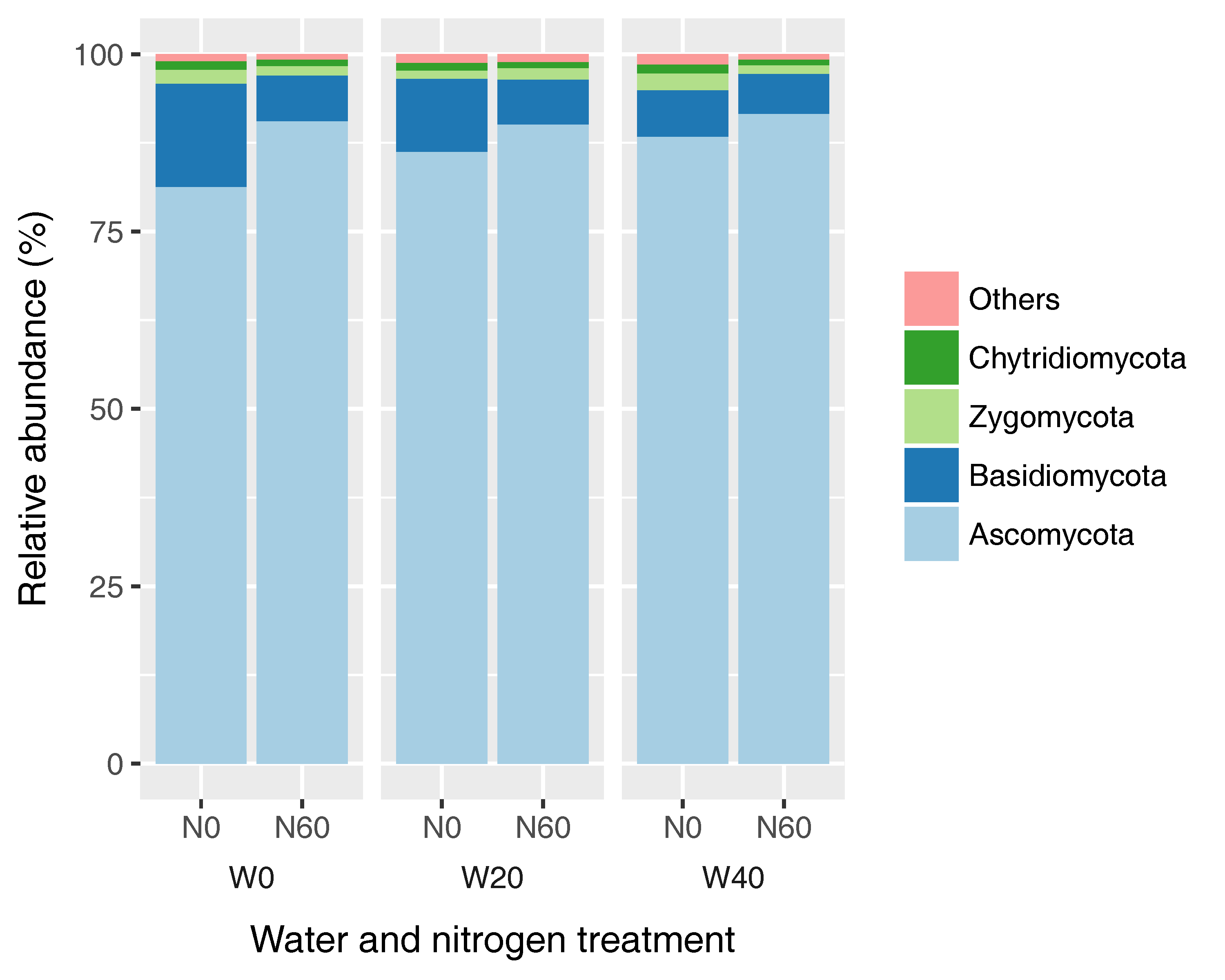


**Figure S4.** Relative abundances of soil fungal orders under different water and nitrogen treatments. Individual orders with a low relative abundance (<1%) were integrated into the “Others” category.


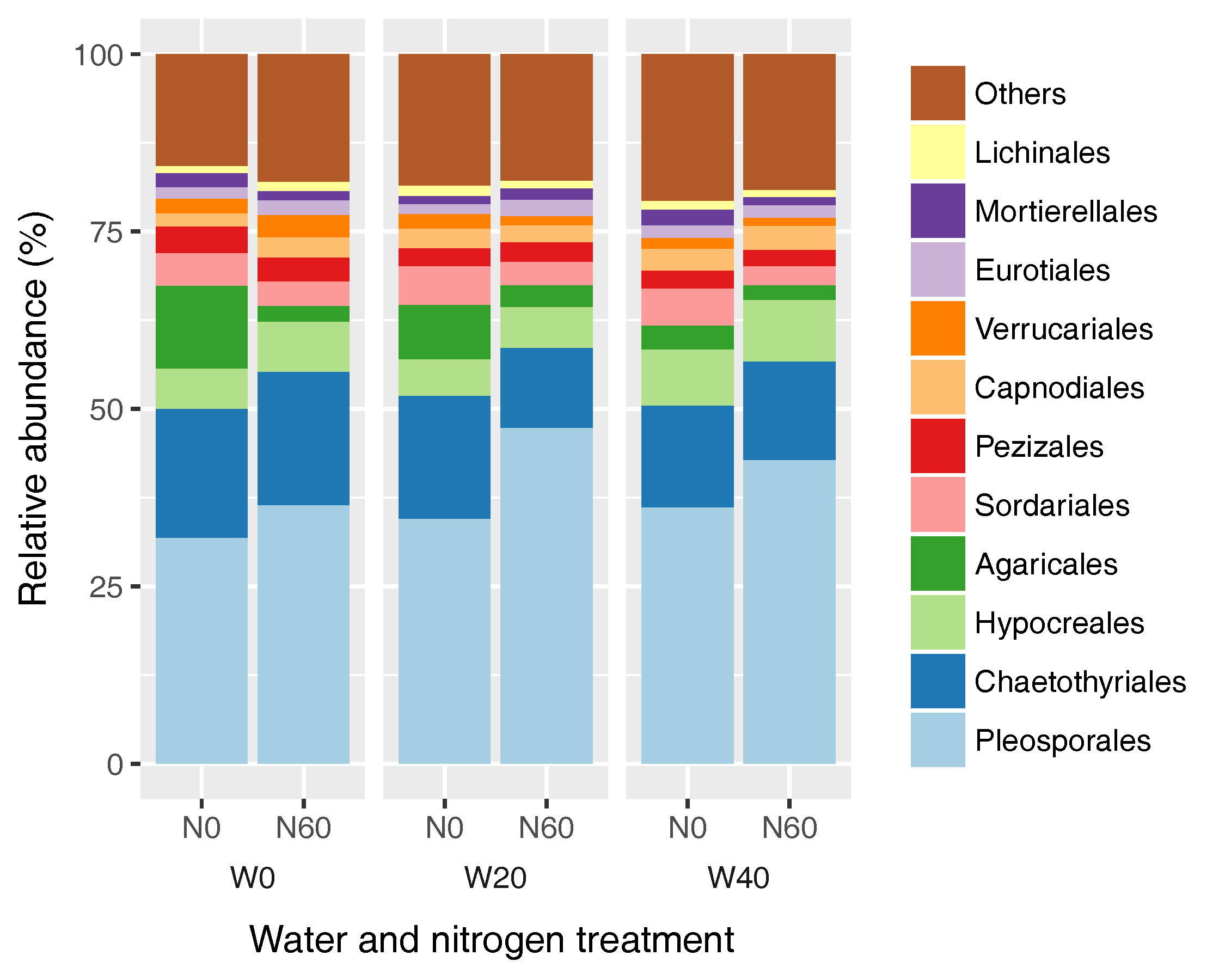

Supplement: Supplementary file 1 [file Data_Sheet_1.DOCX]
